# COVID-19: what is not being addressed

**DOI:** 10.1177/0956247820963961

**Published:** 2021-04

**Authors:** Jaideep Gupte, Diana Mitlin

**Keywords:** community-led, COVID-19, digital infrastructure, disaster response, informal settlements, SDG11, smart cities, urban space

## Abstract

As the number of confirmed COVID-19 cases nears 27 million, there is a rush to answer (what next) and a rush to act (to solve the immediate problems of COVID-19). This paper discusses, with a specific focus on urban areas in the global South, what is missing to date from this response. That includes an identification of things that there are too much of, things that are not being done at all, and things that are unbalanced. There has been an enormous upsurge of academic research papers and opinions on COVID-19. “Technological” and “scientific” solutions tend to overshadow other approaches, even if people know that “social is important”. Based on our analysis to date, our primary concern is that there is too little understanding about the importance of building dialogue, exploring collaboration and co-producing solutions. There is too little understanding as to *why* social and cultural responses are important, and *how* the recognition that they are important can be actioned.

**Figure:**
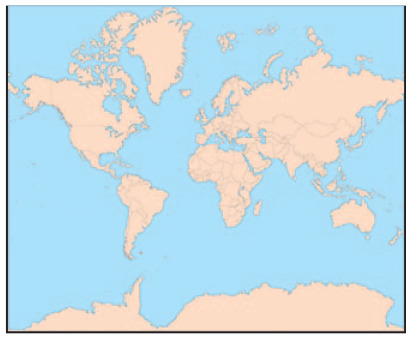


## I. Introduction

Over 95 per cent of all COVID-19 cases globally occur in urban areas.^(1)^ The very nature of pandemics is that they are dependent on the interactions of humans with their environment and with each other. These interactions can become intensified in the built environment, making epidemic control a key consideration in city making. The indirect and long-term impacts are likely to be more pronounced and protracted, requiring more complex responses, in lower- and middle-income countries (LMICs) due to their more limited ability to respond to the risks. Globally, almost one billion people live and work in conditions of urban informality and precarity. Hundreds of millions more living in LMIC cities can just about afford homes with formal services such as piped water, electricity and access to healthcare, but these services are patchy and reliant on deteriorating infrastructure.

While these challenges are location- or place-specific, it is misleading to limit the assessment of the ecology of risk, the direct and indirect impacts of the disease, or the implications for long-term responses, to city limits alone. We recognize that urban systems share a deeply symbiotic relationship with peri-urban and rural contexts, not least through their infrastructural links and dependencies, but also more directly as a result of food systems and patterns of human mobility, and it remains important to take account of these wider contexts. Take the example of the first human-to-human transmission of the coronavirus in Germany, which reportedly occurred in January in a car parts company in Bavaria, when a worker asked a colleague to pass the salt.^(2)^ However, as Roger Keil and colleagues point out, the coronavirus had travelled to this distant Bavarian suburb from the periphery of Wuhan, where 1.6 million cars were produced last year.^(3)^ Hence the spatial analysis of risk has to include the very local as well as the global.

As two urban scholar-activists, we are motivated to write this paper because we are concerned that COVID-19 will exacerbate existing inequalities and absolute deprivation and disadvantage, while fuelling a misinterpretation of the nature of societal relationships that produce and sustain agglomeration. We have observed this happening both through the movement of the epidemic on the ground in the context that we know (i.e. low-income neighbourhoods), and through the ways in which local and national governments, as well as external agencies, are responding to this situation. We take note of a misconception that COVID-19 is *caused by* agglomeration and therefore requires “de-densification”^(4)^ or heightened surveillance^(5)^ in cities – it is not. It is a global *health crisis*, whose impacts on urban populations are exacerbated by debilitated – or outright nonexistent – health infrastructures and, as we expand upon in this paper, the unequal nature of socio-political and economic relationships that categorize the circumstances faced by the lowest-income urban residents. In this context, there are implications for the *local actions* global compacts aspire to in order to achieve sustainable development, as well as for our understanding of urban living itself.

The impacts will be most dramatic where health systems are inadequate, unreliable, under-resourced and mismanaged. While most reported COVID-19 deaths have occurred in Europe and North America, there are numerous concerns about the accuracy of global estimates of both mortality and infection. The data that we do have suggest that Latin America, where the number of deaths surpassed 100,000 in June and the number of infections doubled to 2.2 million in less than a month,^(6)^ and increasingly South Asia,^(7)^ are particularly badly hit. Guayaquil and Rio de Janeiro illustrate why we are concerned. Low-income residents are unable to protect themselves. Overcrowding and lack of access to basic services prevent the implementation of two key measures that populations are being urged to adopt: social distancing and increased hygiene, particularly handwashing. In terms of state interventions, curfews and lockdown make income generation difficult to impossible. Informal traders have been stopped from operating, while formal shops selling food and sometimes other goods are allowed to be open. Formal manufacturing businesses have closed, laying off workers, and subcontracting is not taking place. Low-income households face acute difficulties with limited savings; the need to pay for food, rent and services; and limited access to social protection.

Given what feels to be a rapidly moving set of activities and reversals, we wish to contribute our reflections on current debates. The required speed of our response means that this contribution is not based on a methodologically robust research design carried out in multiple locations in a comparative analysis. Rather, we draw on our research network and our research experience to survey the information available to us, test out emerging observations and lessons, recraft our conclusions and test them again. This means that we are reading, analysing and synthesizing reports from a network of colleagues that has been built up over 20–30 years. Many of these reports are from people we are working with, or have worked with for many years as research collaborators, whose capability and integrity we trust. In other cases, we are using sources of information that we have also worked with for some time, although we may not have worked directly on the same research projects. As we have come to know them, we have found these sources to be accurate as providers of information, and their analysis and conclusions are verified by other information and by our colleagues who know that context well.

A typical day over the months since early March brings at least five notifications of new reports, blogs and webinars. We have each been checking these sources regularly, scanning them to understand what is going on in towns and cities across the global South, and the responses there to the health and economic crises. These reports have adopted a variety of research methods including ethnographic reporting, analysis of medical data, community monitoring and a host of other techniques. All of these sources were published between 18 February 2020 and 12 August 2020. Tables 1 and 2 summarize the set of blogs, grey literature and media sources we have consulted; a complete list is available in the online supplement. We have also checked these findings against peer-reviewed academic papers about pandemics, recent and longstanding, and through discussions with other scholars, particularly those in the medical profession. Moreover, we have not been passive listeners. We have ourselves lectured or presented on these topics, written blogs and articles, and provided policy advice over the past months. Indeed, our ongoing research activities have also directly or indirectly engaged with COVID-19. We are deeply appreciative of the multitude of activities and accounts that have been sent to us, the emerging understandings that have been shared with us and the invitations that we have received. The conclusions here draw on these sources, as understood over the last three months, captured through conversations, blogs, reports, webinars (both presentations and discussions) and news stories.

**Table 1:** Types of blogs, grey literature or media sources consulted

Type of blog, grey literature or media source	#
Blog – personal	7
Blog – institutional	14
Institutional grey literature	14
News/media	24
TOTAL	59

**Table 2:** Geographic and thematic spread of blogs, grey literature and media sources consulted

Primary geographic focus	Early/direct health impacts	Secondary/ societal impacts	State response	Civil society response	Regional totals
Asia	1	6	2	5	*14*
Africa	3	13	4	6	*26*
Latin America and the Caribbean	1	7	1	0	*9*
Multi-country/multi-region	2	6	2	1	*11*
Other	1	11	0	2	*14*
Totals	8	43	9	14	

NOTES: The numbers in Tables 1 and 2 refer to all such sources cited in the footnotes in this article, but do not include the peer-reviewed studies cited.

The next section outlines our key concerns with COVID-19 responses in LMICs and associated discussions. The subsequent section explores the failure to identify and understand what is taking place in towns and cities in the global South, and the potential consequences of this lack of understanding.

## II. Key Concerns

As we write this in August 2020, the number of confirmed COVID-19 cases globally stands at over 26 million, with 824,162 deaths thus far.^(8)^ *“By one estimate, the COVID-19 literature published since January has reached more than 23,000 papers and is doubling every 20 days”*.^(9)^ This constitutes one of the largest upsurges in research on any topic. Technology-based responses to COVID-19 seem to play a particularly dominant role. One commentator groups the digital responses to the pandemic into five categories:^(10)^ solutions for effective and efficient contact tracing, responding to the need to track the pathogen exchange faster than traditional systems of disease reporting; testing and disaster responder capacity to improve, adapt or invest in medical devices, tests and protective gear; early warning and surveillance systems to better understand the pathogen and monitor the outbreak; quarantine and social control as important elements of the human side of a pandemic response; and technical advancements in vaccine, mitigation and treatment research.

We find there is a critical gap between the technological solutions being suggested and whether they contribute to inclusive, resilient and sustainable responses from the perspective of economically and socially disadvantaged urban residents. We see that tech-based responses are often based on uncritical and unnuanced techno-utopian understandings of what are deeply unequal relationships.^(11)^ At the same time, techno-utopian narratives are an “easy sell”, particularly to those who do have access to digital infrastructures and therefore stand to benefit from technological interventions, and they serve as an illusory alternative for meaningful local action.

App-based track and trace “solutions” proposed in various countries are an illustration of this. Compare for example, the Aarogya Setu mHealth app^(12)^ promoted by the government of India,^(13)^ and the increasing unease with its use and impact on digital freedoms.^(14)^ These interventions highlight the need for an engagement with issues of inequality and social relations, themes of concern to the social sciences. They mistakenly assume equality of access to digital infrastructures, which in fact are gendered and unequal: across low- and middle-income countries, women are 20 per cent less likely than men to own a smartphone or use mobile internet.^(15)^ The gender gap is exacerbated in low-income areas; a study of informal settlements in Nairobi, Kampala, Lagos, Yaoundé, Maputo, Cairo, Bogotá, New Delhi, Jakarta and Manila revealed women are about 50 per cent less likely to be connected to the internet and a third less likely to use a smartphone to connect, compared to men in the same age group with similar levels of education and household income.^(16)^

Track and trace approaches assume that the disease is spread through contact resulting from voluntary movement alone and that people do not live in overcrowded dwellings, and can therefore be isolated once identified. This completely disregards the *compulsory* nature of the economic and sociocultural relationships that force people to continue living and working in precarious locations despite the risk of contracting or spreading the disease. Track and trace approaches also severely misunderstand the magnitude of the involuntary movement of people often forced by authorities or landlords who, at least in the initial stages of lockdown, continued evictions unabated.^(17)^ The reflections of Alice Wanini, a community health volunteer working in Nairobi’s Mukuru Village (home to 100,500 households), are captured in a blog series by the Muungano Alliance, a civil society group in Kenya that is affiliated to the transnational network SDI.^(18)^ This is a neighbourhood in which. . .

“. . .space is a luxury- and social distancing is nearly impossible, both within the households and on the narrow streets around them. [Alice] tells of an incident that recently transpired where a water boozer [bowser or water tanker] supplying free water in the community, brought hundreds of residents scrambling for access to free water. In an area notably known for its erratic water supply, the free supply of water to support regular hand washing and other household usage foresaw residents crowd at the water supply area each aiming to get their water cans full with little care to prioritize social distancing.”^(19)^

Earlier research details the high densities and lack of services that residents face in this neighbourhood.^(20)^

These challenges run deeper than in relation to specific technologies. COVID-19 demands and has secured a wealth of research projects.^(21)^ It appears that many of those with solely medical interests have little understanding of the realities of people living in informal settlements,^(22)^ where low wages and relatively high costs of housing and essential services mean that cash-at-hand and savings are inadequate.^(23)^ Measures to control population movements and their consequences for income generation have therefore triggered an economic emergency. Here, we recognize the need to look beyond *“the immediate health consequences of the pandemic”*, particularly as these *“begin to be superseded by the impact of public health containment measures”*^(24)^ – notably through the inability of those subject to these measures to buy essential goods and services. Social and economic interdependencies skew health and wellbeing outcomes, but in our opinion, and as we elaborate upon in later sections of this paper, are likely to be underestimated by those without an engagement with the social sciences.

Furthermore, the lack of literacy and/or the deep suspicion of formal processes on the part of many low-income residents mean that preferred “modern” approaches to ethics as developed in the global North are not effective in securing the desired ethical response. Northern ethics research requires informed consent and documentation to show that this has been secured. Typically, this is a form signed by the interviewee. People who are illiterate are asked to listen to a reading of the form and indicate their agreement in some way. However, it is rare that allowance is made for their fear related to officialdom. If their vulnerabilities are recognized, then it may be suggested that this group is left out of the sample. However, excluding those who are illiterate and/or who are unable to take part for these reasons is likely to bias the results, as the same people may have significantly lower incomes. Researchers must engage with those who are familiar with local contexts, and those who have been trained in appropriate research methodologies.

We believe that societal relations are key to addressing COVID-19 – and that these relations have implications for technology and science. Solutions have to be locally owned for them to have any chance of success, as shown by Matt Nohn^(25)^ in his analysis of responses to Ebola in Monrovia. This level of behavioural change cannot be imposed from above; the required modification of everyday practices has profound implications for all relations including the most intimate. In addition, strong local engagement will enable generalized solutions to be modified so that they are effective within local realities. Such modifications to designs are essential to – and as important as – local ownership. The co-production of responses is essential to secure the required modifications to designs and their effective adoption. Too little attention has been given to this. This is the context in which we understand *Al Jazeera*’s report from 5 June 2020 that there have been 15 killings in Kenya to enforce the COVID-19 related curfew.^(26)^

In sum, there is an oversimplistic approach to the urban context by “non-urban” professionals and academics. Too little attention is given to the multiplicity of ways in which the “urban” nature of settlements and livelihoods influences what is possible in terms of responses to the health and economic emergencies. There needs to be a substantively greater consideration of what it means to seek to intervene in an urban location. We put forward several points in this regard:

***Agglomeration and the nature of societal relationships*:** It is critical to recognize the nature of social relations, the intensity of – and interaction between – government agencies, and the interconnections between multiple and cascading risks and structural disadvantage. This includes a recognition of the nature of relations between urban residents, the intensity and diversity of state agencies, the creativity of people through interactions and opportunities for capability development, and the dynamic nature of (some) urban contexts.^(27)^ We take note that grassroots organizations are doing much to address local needs. Isabel Duque Franco et al.^(28)^ document and analyse over 200 community initiatives to address local needs related to the COVID-19 pandemic across Latin America. Thirty-seven per cent are related to food security and 34 per cent to public health. A further example is provided by a community leader from Mumbai who discusses the ways in which members of Mahila Milan (a collective of women’s savings groups) are helping to distribute food donated by local politicians, focusing on those who do not have ration cards and therefore cannot benefit from state support.^(29)^ Another survey of 321 “slum leaders” across 79 informal settlements in two north Indian cities (Jaipur and Bhopal) finds that community leaders in these settlements have persisted in their problem-solving roles during the pandemic and relied upon their informal authority and political networks to request urgently needed government relief.^(30)^ The study also finds however, that community leaders *“are not equally positioned to secure information, make claims, and command government responsiveness in the context of the pandemic. . . leaders that are more deeply entrenched in partisan networks also appear to be more likely to contact, and be contacted by political elites, as well as report having received assistance to pandemic-time claims”*.^(31)^ This raises concerns regarding the inclusiveness and fairness of these efforts. In the Philippines, the Homeless People’s Federation did not wait for the government but immediately bought supplies for the neediest households in the neighbourhoods in which they had active organizations. while also working with state agencies providing welfare and relief.^(32)^

These local efforts notwithstanding, in our opinion the nature of urban organizations – and the potential for resistance and collaboration they display – remains understated in present pandemic responses, requiring a recalibration of interventions towards co-producing solutions to COVID-19.

***Agglomeration and the commodification of labour*:** Low incomes make it impossible for many living in towns and cities of the global South to secure adequate accommodation. Poorly informed government strategies to reduce poverty lay too much emphasis on cash incomes and not enough on the social wage (i.e. access to affordable basic services including water, sanitation, energy, health and education). Without access to these public services, households cannot afford safe and secure housing. There is insufficient information on the costs of adequate access to basic services. While community groups have sought to improve access to the services required for health,^(33)^ without state investment this cannot be done effectively and at the scale required.

As noted in the following section, there have been multiple reports that low-income households – embedded in economic and spatial informality – have not received social protection. Cash transfers are seen as the key source of support (regardless of whether they are actually received). As analysed by WIEGO, a transnational network working with women employed in the informal economy, government measures have been partial.^(34)^ Insufficient attention has been given to the heightened vulnerability profiles due to multiple and cascading risks drawing residents further into destitution – and this may be true even for those who were just about surviving before the pandemic. In Peru, for example, informal workers have been denied access to government emergency COVID-19 grants because the government does not have lists of those in extreme poverty in urban areas, as it does not consider there to be extreme poverty in these areas.^(35)^ Insufficient consideration is given to urban social assistance going beyond cash transfers, *“to focus on generating jobs (especially for young people and women) and to link low-income urban residents to basic services such as health care, through subsidies, vouchers, or case management”*.^(36)^ A focus on cash income is insufficient for understanding the complex survival struggles of the urban poor. For centuries, people have made trade-offs, staking their health and livelihoods on living and working in precarious conditions. These trade-offs not only underpin their risks and vulnerability to disease in the present situation, but also speak to a much deeper history of marginalization and exclusion. There is a need to draw on the longstanding experiences of these trade-offs to better understand the ongoing health and economic pressures, and to prepare for recovery.

WIEGO also highlights the need for state support for economic recovery for informal sector workers.^(37)^ What is telling is that, despite their comprehensive engagement with government measures, they are only able to identify three countries (Burkina Faso, Jordan and Uruguay) that exemplify the measures that might be taken. There is a real fear that lockdown measures will exacerbate inequality. For example, in Peru, supermarkets have been allowed to remain open and sell a range of essential and non-essential goods, but the only informal workers allowed to remain trading are those selling essential goods.^(38)^

A key constraint to effective response is identifying and utilizing the appropriate and adequate space for pre-emptive measures (e.g. construction of appropriate wash sites with drainage, strategies to isolate patients who have tested positive), responses (e.g. routes to and from hospitals and parking spots for ambulances; transfer of unwell or indisposed patients from residences), distribution of aid (food, water or other essential distribution), and recovery interventions (e.g. disinfection or sanitization of spaces; opening-up of markets; staggered lifting of other lockdown policies). The lack of adequate consideration of informal vendors who are dependent on street spaces is just one example; WIEGO reports on the devastating consequences of this lack of understanding for the livelihoods of these low-paid street workers.^(39)^ Early experiences from news websites show that the demand for space can change rapidly: the sudden crowds of migrants gathering at the Anand Vihar bus station in Delhi (on 28 March),^(40)^ long queues developing suddenly as food aid was distributed in Dharavi, Mumbai (on 6 April),^(41)^ and similarly in Alexandra township, Johannesburg (on 28 April).^(42)^ Moreover, space considerations vary by location as well as by gender, age and other socioeconomic markers that influence risks and vulnerability profiles. The visualization of informal settlements in many COVID-19 discussions, however, is of homogenous high-density inner-city shacks, with insufficient attention given to lower-density settlements (more likely to have urban agriculture) that may also face health and economic emergencies. Furthermore, decisions over the use of space are being carried out in real time and usually from a distance in compliance with lockdown. Given the short supply of open space in dense urban areas, intervention sites are being used for multiple actions simultaneously. However, not enough resources are being devoted to rationalize clearance times, to formulate patterns for safe distancing, and to manage perimeters. The onset of rains will change drainage and access patterns, while soil conditions, especially in informal settlements, will increase the risk of other water-related infectious disease, such as malaria, dengue and cholera.^(43)^ Proximity to other vulnerable areas or hotspots is also an important consideration, though it has been understated in determining the efficacy of response spaces and strategies, including those involving the eventual easing of lockdown policies.

***The potential within urban contexts*:** The rapid response to the need for masks – in multiple countries – is one example of the potential processes of production, consumption and creativity that exist. The *Guardian*’s report on mask making in multiple locations by diverse groups illustrates this,^(44)^ although it may not give sufficient attention to individual entrepreneurship. But this requires an understanding of this context, a sensitivity to the way in which governments intervene, and attention to the unintended and unanticipated consequences of such interventions. In Kenya, the Muungano Alliance, in recognition of the impossibility of informal settlement residents isolating at home, has produced maps of informal settlements indicating places that are appropriate for isolation centres.^(45)^ In Lima, street vendors faced a strict curfew introduced on 16 March, as well as state actions against informal workers and a lack of access to government emergency grants. They responded by negotiating with local government for the return of confiscated goods, launching a media campaign to demonstrate their willingness to comply with hygiene measures, and articulating their potential contribution to a safe and secure food supply within a more equitable city.^(46)^ The wealth of grassroots responses to COVID-19 is elaborated by the International Institute for Environment and Development (IIED), which has drawn on experiences from across its partners in the global South, who provide evidence of local groups stepping in to reduce health risks and provide emergency access to food and hygiene.^(47)^

## III. Consequences

As a consequence of the failure to adequately contextualize, responses are deficient in three ways.

First, there is a tendency to focus concerns on issues of density without adequate attention to lack of services and overcrowding. Both the press and the experts have recognized that urban areas are particularly at risk as the disease is spread by close contact, to which social distancing is the solution. The urbanization focus of COVID-19 is exemplified by the *Financial Times* (in its free-to-read coronavirus updates^(48)^), which has been reporting “excess deaths” in 15 urban centres, including five from the global South. The crude aggregation of population density and impact pays insufficient attention to the circumstances under which risk and vulnerability occur. As elaborated by Harvard University Professor Mary Bassett in the *New York Times*^(49)^ for the US, the context is everything. This is a context in which poverty leads to residential overcrowding and lack of access to adequate levels of the basic services required for hygiene. Density itself is not the problem; it is the overcrowding that is a result of poverty and the lack of infrastructure and services from state neglect.

The climate emergency adds to the need for clarity on this point. There has been a recognition that a successful transition to a low-carbon economy is facilitated by more compact cities, and that densification is a solution rather than a problem. However, as noted by Justin Visagie and Ivan Turok,^(50)^ densification requires careful planning, especially given the implications of the irregular plots that are frequently associated with informally developed urban neighbourhoods. Infrastructure networks and housing need to be planned both to be affordable and to be consistent with overall development of the town or city.^(51)^ In the current context, de-densification is viewed with trepidation by those who fear that COVID-19 will be associated with evictions legitimated through this rationale. See, for example, the response of South African civil society when their government highlighted the potential of “de-densification” as a response to COVID-19.^(52)^

The *Financial Times* reports that Guayas Province in Ecuador has had 12,200 excess deaths, which is 248 per cent of what would have been anticipated.^(53)^ The vulnerabilities of the population in the provincial capital, Guayaquil, are elaborated by University of Manchester Emeritus Professor Caroline Moser and architect Olga Peek in an IIED blog post, and relate both to globalization (travel to and from Spain, Italy and the US in particular) and poverty that puts healthcare out of reach.^(54)^ In response to overcrowding at home, young people have been on the streets, potentially adding to risks.

In Nairobi, the scale of deprivation in informal settlements is evident. In Mukuru, for example, only 1 per cent of residents have access to a private water tap and private sanitation. The lack of provision means that a public tap serves an average of 234 households, and there is one public latrine for every 547 households.^(55)^ This study also documented just how expensive it is to be “poor” and the premium paid to access housing and informal services – not a new story in any respect. More recent research has pointed to the reality that this premium may be related not just to the higher cost of informal services but also to the adverse circumstances under which low-income households access formal services.^(56)^ Rigorous community monitoring has shown that despite these adverse conditions, there have been relatively few cases of COVID-19 in Nairobi, Kisumu and Nakuru.^(57)^

The impacts of the responses to COVID-19, as with historical responses to public health crises such as Ebola (in 2014),^(58)^ H1N1 (in 2009) or SARS (in 2003)^(59)^ – or even further back historically, the cholera pandemics in cities (for example in London and New York in the mid-19th century)^(60)^ – are filtering through to built environment design, planning and building processes. In the initial weeks of the COVID-19 pandemic, much accusatory attention was directed towards high-density urban areas and mobilities within such areas.^(61)^ Subsequently, however, *“the contemporary processes of extended urbanisation, which include suburbanisation, post-suburbanisation and peri-urbanisation”*, have also been linked with *“increased vulnerability to infectious disease spread”*.^(62)^ Given this, Creighton Connolly writes, *“governance is a more important factor than density in determining the severity of outbreaks”*.^(63)^ But there is no consensus as yet on the practical steps required to rethink the material production of the city, nor on how managing density (and overcrowding) and installing new ways of transportation, relate to the vulnerabilities of the lowest-income urban residents living and working in precarious sites. How do we prevent a response driven by environmental health imperatives that is fundamentally hostile to informal dwellers, condemning them because of their poverty? The required response has to prioritize essential investment in basic services and the provision of affordable housing so as to assist low-income households to secure access to the services and housing that they need for good health and wellbeing. “Affordable” in this context cannot be synonymous with “low-cost” unless low-cost is indeed affordable to the lowest-income households.

Second, the actions and messaging are too frequently top-down, with little attention being given to questions of local ownership. In Kenya, the Muungano Alliance have established a monitoring system^(64)^ in settlements where their membership is strong, to ensure that the government and communities have accurate information. On 20 June 2020, they collected data from 193 community leaders (youth, community, health workers and village elders) from 42 villages in three cities (Nairobi, Nakuru and Kisumu). Their data highlight that the areas with the most restrictions are not those with the highest reported rates of infection. Specifically, 40 per cent of informants reported cases of COVID-19 in Mathare, but informants for this area were less likely than those in other areas to report the presence of isolation centres, social distancing and restrictions on movement. Pressure from the communities has meant that the Alliance is sending the information back to the key informants with summaries of the situation along with updates on both the situation and what is being provided nationwide.

Going forward, the ability of grassroots organizations to engage with digital information is an opportunity to build new relations between responsible state agencies and organized community groups. The significance of co-productive responses, and the need to ensure strong local ownership, means that this should be explored. However, attention also has to be given to the digital divides, women’s relative lack of access to mobile phones, and the risk that some of the older vulnerable groups in the population will not be equally able to represent their needs and interests.

Third, while recognition is being given to the economic emergency, actions do not acknowledge the depth of the crisis that is facing the urban poor. It is evident that considerable numbers of informal workers and enterprise owners are either unable to sell anything at all because they are forcibly closed, or are selling considerably less than usual due to reduced hours and/or consumers having less to spend.^(65)^ In Nairobi, for example, a curfew from 19:00 to 5:00 has considerably reduced the numbers of clients and customers, and a complete lockdown in areas such as Eastleigh has meant that domestic workers have lost their incomes – with obvious knock-on effects within, in this case, the adjacent informal area of Mathare.^(66)^ The impacts are similar in middle-income countries such as South Africa.^(67)^ However, while governments promise income support and emergency cash transfers, many households living in informal settlements have not benefitted from this provision. In Nigeria, Justice and Empowerment Initiatives have worked with the Nigeria Slum/Informal Settlement Federation and the Physically Challenged Empowerment Initiative to track the neglect of those living in informal settlements and their inability to either earn a living during lockdown or access government funds. A survey of 383 leaders in 114 communities found that *“78 per cent reported people are unable to meet basic needs. Meanwhile, the vast majority of urban poor communities (85%) reported government-provided “palliatives” intended for the vulnerable had not reached them.”*^(68)^ Similar findings are evident in Brazil where, for example, low-income favela residents in São Paulo are not receiving the monthly emergency basic income payment (worth US$ 115), despite the shutdown by the city authorities of informal trading on 15 April 2020.^(69)^

Longer-term impacts for low-income residents are likely to be severe, particularly for those engaged in informal livelihoods. The economic consequences of lockdown are already evident in testimonies from those living and working in the informal sector. An early analysis of Dhaka, Bangladesh is provided by Stuart Rutherford, a longstanding expert in financial services for low-income households.^(70)^ The lockdown here began on 26 March 2020 and lasted until 30 May, although it was progressively less actively respected and enforced. Data from 60 individuals who keep financial diaries (a process ongoing since August 2017) showed a significant collapse in total flows of money. In April 2020, the first full month of the COVID-19 lockdown, total money exchanged (inflows and outflows) aggregated across all 60 diarists fell to 1.62 million Bangladeshi taka (US$ 19,000). For comparison, the average equivalent amount of money (from August 2017, when the diaries began) was 5.49 million taka (about US$ 65,000). Average household expenditure of 500 taka a month was financed by income (160 taka), loans (150 taka) and savings withdrawals (100 taka), with the balance being met by gifts and a small payment of 20 taka for the completion of the diary. However, these average figures are deceptive as one-third of all expenditure was made by one family that included two government employees. The remaining families spent four times more than they received in income during April, with 72 per cent of non-construction-related expenditure^(71)^ being spent on food. Dramatic falls in income for low-income families in Brazil are also reported by *RioOnWatch*, which regularly updates readers on the challenges faced by favela residents. In this case, *RioOnWatch* draws on a national survey to report, *“Four out of five favela families (80%) across the country are living on less than half the income they received prior to the pandemic, the study found. Of those 80%, more than a third reported losing all of their family income, while only 9% of the total experienced no change in income or only a minor drop.”*^(72)^ One result of this loss of income, combined with a lack of social protection, is that low-income residents are forced to find work despite the risks to themselves and their communities. John Taylor, chief technical advisor for the Food and Agriculture Organization (FAO) in Bangladesh, reports that some low-income workers in Dhaka have been working as “rogue rickshaw drivers”.^(73)^ He notes that households and communities have turned to urban agriculture as a way to secure access to food.

Furthermore, national strategies have required a homogenized (and often brutal) enforcement of order under lockdown. This has led to a heightened incidence of violence, fear and anxiety, experienced particularly by those who were already targets of violent evictions or enforcement of city-planning and state practices, and who suffered sociocultural marginalization and stigmatization as a result prior to the pandemic. As noted by the UN High Commissioner for Human Rights, there has been a troubling increase in cases of police brutality and rights violations under the cover of exceptional or emergency measures.^(74)^ An inability to rely on informal arrangements with non-state actors, including illegal and extra-legal groups, has meant the police are hindered in their ability to provide safety and security during the COVID-19 pandemic. On the one hand, the police are failing to protect communities from criminal gangs, where they pose an increased risk.^(75)^ At the same time, they have hampered aid efforts when these have come from non-state or criminal groups.^(76)^ City police are tasked with placing physical constraints to stop people from reaching their places of work,^(77)^ while the closure of markets, demolition of informal shops^(78)^ and prohibition of street vending have prevented people from earning a living.^(79)^ There is also a notable increase in domestic violence, violence against children, gender-based violence (GBV) and antisocial behaviour.^(80)^ We note that in these examples, police and other security services were described as overburdened. Trust was noted to be low between communities and enforcement officials, who have been exposed to high levels of violence, service delivery protests and criminal activities that have existed since before COVID-19, while at the same time being exposed to COVID-19-related risks themselves.^(81)^ Maintaining law and order has proven doubly complex given that legal and procedural practices have also needed to adapt extensively and repeatedly to situations of social distancing and lockdown, as exemplified in South Africa.^(82)^ In most LMIC contexts, police personnel, particularly those holding lower ranks, have limited opportunities to access mental health and other wellbeing support. It is under these difficult circumstances that city police forces have become central to national COVID-19 response strategies. They are required to enforce regulations that are often unpopular in public, private and commercial spaces, in lived environments as diverse as informal settlements and gated communities, including their own poorly serviced living quarters, as well as online in response to new and increased digital activities.

## IV. Conclusions

As authors of this paper, we remain deeply sceptical that the pandemic, and the policy or programmatic interventions in response to it, will trigger the sort of system-wide rethink required to uplift urban futures, particularly for people who live and work in conditions of urban informality and precarity. On the contrary, the most likely outcome is an ossification of pre-existing inequalities, persecutions and structural failures. Yet again, we see “the local” placed as the frontline of crisis response. And yet again, the frameworks that guide our collective thinking seem incapable of adequately devolving political authority with adequate resources to this level. Without a meaningful and sustained political shift towards the local, crisis response remains debilitated. We despair as we note parallels with the plague epidemic in Bombay all the way back in 1896, when colonial policies aimed at supressing the disease showed explicit class bias against the urban poor.^(83)^ Those experiences *could have* triggered a new way of city planning, involving a rethinking of urban service infrastructures, and a re-strategizing of crisis responses, to engage the politics and negotiations of agglomeration. More than a century later, however, Mumbai finds itself in the same position.

What then is the potential to see the shock of COVID-19 as an opportunity? There is – it seems – a new attention to towns and cities in the context of the COVID-19 pandemic. For example, we see this in Nairobi, where the national government has committed to informal settlement upgrading. In August 2017, the government declared a Special Planning Area with two years to prepare and deliver an integrated plan. That period has now been extended for a further two years. What is exciting in Mukuru is that infrastructure improvements in water and sanitation, roads, drainage and electrification have now been finalized, and implementation by Nairobi Metropolitan Services began in May 2020.^(84)^ And the Kenyan president announced upgrading plans for both Korogocho and Mathare on 18 March 2020. Meanwhile Nairobi Metropolitan Services announced the suspension of all existing development plans in four further informal settlements in Kibera on 26 May 2020, in order to enable a more participatory integrated approach.^(85)^ On 14 February, the City Hall (Nairobi County) and the Ministry of Transport and Infrastructure announced their intent to scale up the upgrading of Nairobi’s informal settlements with a Special Planning Area for the neighbourhoods around Nairobi Central Station. COVID-19, then, has been associated with an acceleration of plan implementation in Mukuru and significant new upgrading initiatives across Nairobi.

Once new external agencies and their interventions are better informed about the urban context and more successfully embedded within urban institutions (both formal and informal), the situation might be recognized as an opportunity. This requires addressing the unequal socioeconomic and political relationships that have come to characterize our cities with a set of reforms that have broad ramifications and that can lead to a realization of the *local* objectives of key global compacts such as the Sustainable Development Goals. We think that closer attention to such reforms will be helpful as long as it recognizes the insights of the social sciences and the knowledge created and owned by local communities. There is an urgent need to invest in the infrastructure required to make low-income housing safer, especially dwellings in informal settlements. And there is a need to support better-quality housing, through access to more affordable improvements. At the same time, for an effective response to the health emergency, there is a need for residents, community health workers and/or volunteers, and the health authorities to work together. It is critical that those who are knowledgeable about effective urban development approaches are asked to share their expertise. Successful interventions are those that recognize the need for targeting financial support, and devolving authority to respond to the municipal (even sub-municipal) level, while at the same time proceeding based on knowledge about economic and spatial informality and building on the efforts, knowledge and capabilities of a range of local agencies, particularly the grassroots organizations that do so much to address local needs.

## Supplemental Material

gupte-mitlin-supplement – Supplemental material for COVID-19: what is not being addressed Click here for additional data file. Supplemental material, gupte-mitlin-supplement for COVID-19: what is not being addressed by Jaideep Gupte and Diana Mitlin in Environment & Urbanization

## Notes

1. UN-Habitat (2020).

2. Schmitz (2020).

3. Keil et al. (2020).

4. For example, in March 2020, the South African Minister for Human Settlements, Water and Sanitation identified 29 areas, in Gauteng, KwaZulu-Natal, Western Cape and Eastern Cape, for de-densification. See *BusinessTech* (2020).

5. See the example of cities in Brazil employing mass surveillance to monitor urban mobility in Peron et al. (2020).

6. Jorgic (2020).

7. *South Asia Monitor* (2020).

8. See https://ncov2019.live, accessed 26 August 2020.

9. Brainard (2020).

10. McDonald (2020).

11. See the example of an expanded surveillance state in China as summarized by Oxford Analytica (2020).

12. Aarogya Setu is described on the Google Play Store as *“a mobile application developed by the Government of India to connect essential health services with the people of India in our combined fight against COVID-19. The App is aimed at augmenting the initiatives of the Government of India, particularly the Department of Health, in proactively reaching out to and informing the users of the app regarding risks, best practices and relevant advisories pertaining to the containment of COVID-19.”* See https://play.google.com/store/apps/details?id=nic.goi.aarogyasetu and https://www.mygov.in/aarogya-setu-app, accessed 26 August 2020.

13. Garg et al. (2020).

14. Christopher (2020).

15. GSMA (2020).

16. World Wide Web Foundation (2015).

17. See, for example, the Thompson Reuters Foundation’s report of evictions in Nairobi. Bhalla (2020).

18. See http://knowyourcity.info, accessed 26 August 2020.

19. Gojobe et al. (2020).

20. Mukuru’s population density is reported as being 108,128 people per square kilometre in Sverdlik et al. (2019). And the lack of services is reported in Corburn et al. (2017).

21. See the funding tracker of the UK Collaborative on Development Research (UKCDR) and Global Research Collaboration for Infectious Disease Preparedness (GloPID-R), available at https://www.ukcdr.org.uk/funding-landscape/covid-19-research-project-tracker, accessed 26 August 2020.

22. This finding comes from discussion between medical academics at the University of Manchester and one of the authors with respect to a research project: specifically on how the sample might need to be adjusted to take account of different social categories, and how the questions need to be framed given local realities. These realities lie behind the formation of the online Social Science in Humanitarian Action Platform (https://www.socialscienceinaction.org/about), accessed 26 August 2020. See also Wilkinson (2020).

23. Mitlin and Satterthwaite (2013).

24. Lambert et al. (2020), page e312.

25. Nohn (2017).

26. *Al Jazeera* (2020).

27. Fraser et al. (2017).

28. Duque Franco et al. (2020).

29. Manivananan Devandra (2020).

30. Auerbach and Thachil (2020).

31. Auerbach and Thachil (2020), pages 32–33.

32. Carampatana and Tuazon (2020).

33. Duque Franco et al. (2020).

34. WIEGO (2020a).

35. Ogando and Abizaid (2020).

36. Cuesta et al. (2020).

37. WIEGO (2020b). Also see a blog post written by WIEGO staff: Alfers et al. (2020).

38. Ogando and Abizaid (2020).

39. Balbuena and Skinner (2020).

40. Ashirwad Mahaprashasta and Srivas (2020).

41. *Mumbai Mirror* (2020).

42. *Reuters* (2020).

43. See for example outbreaks during the COVID-19 pandemic of malaria in Zimbabwe [Chingono (2020)]; dengue in the Caribbean and Central America [PAHO (2020)]; and cholera in Uganda [Taremwa (2020)].

44. Hodal (2020).

45. Muungano wa Wanavijiji (2020a).

46. Ogando and Abizaid (2020).

47. IIED (2020).

48. *Financial Times* (2020).

49. Bassett (2020).

50. Visagie and Turok (2020).

51. Visagie and Turok (2020).

52. Ntseku (2020).

53. *Financial Times* (2020).

54. Moser and Peek (2020).

55. Corburn et al. (2017).

56. Mitlin and Walnycki (2019).

57. Muungano wa Wanavajiji (2020b).

58. For example, Wilkinson and Leach (2014).

59. See for example Ali and Keil (2008).

60. Glaeser (2020).

61. See for example Rosenthal (2020).

62. Connolly et al. (2020).

63. Connolly (2020). Also, see for example Klaus (2020).

64. Muungano wa Wanavijiji (2020c).

65. See, for example, Know Your City TV (2020), and further local accounts in JEI (2020) – for example, Boglo Kehinde’s discussion of the problems of making a living in the lockdown. Also see Waithaka (2020).

66. See Shehu Isa’s testimony on begging and the lack of monies donated in JEI (2020).

67. Oliver (2020).

68. PCEI et al. (2020).

69. JEI (2020); Veloso (2020).

70. Rutherford (2020).

71. Construction-related expenditure is that spent on building and repairing houses.

72. The survey included 3,321 remote interviews, conducted between 19 and 22 June 2020 with residents of 239 different favelas in all 26 Brazilian states. Interviewees were chosen from a preselected sample pool to provide a diversity of region, age and occupation. See Maggiola (2020).

73. Taylor (2020).

74. OHCHR (2020).

75. JEI (2020), Mohammed Zanna’s contribution.

76. Dudley (2020).

77. Goñi (2020).

78. See https://www.justempower.org (accessed 4 September 2020) and @justempower on Twitter for updates on this in Lagos, such as the demolition of shops in Ijora Badia on 30 May 2020.

79. See, for example, this report from Uganda: Hayden (2020).

80. See for example Deb Roy (2020); GBV Guidelines (2020); Batha (2020); and Graham-Harrison et al. (2020).

81. See for example, on the status of policing in India, Common Cause and CSDS (2018).

82. For example, see the various regulations and guidelines, and their amendments in South Africa: South African Government (2020).

83. Kidambi (2004).

84. Kinyanjui (2020a).

85. Kinyanjui (2020b).
