# Supplementary material for: COVID-19: what is not being addressed
Source: Environ Urban. 2021 Apr;33(1):211–28. doi: 10.1177/0956247820963961 (PMC7554414; doi:10.1177/0956247820963961)
Supplement: gupte-mitlin-supplement – Supplemental material for COVID-19: what is not being addressed [file gupte-mitlin-supplement.pdf]

COVID-19: what is not being addressed

Online supplementary information

Underlying data for Tables 1 and 2

Title	Author(s)/publication	Date of publication	Type [blog/opinion – institutional; blog – personal; news/media; magazine; institutional grey literature]	Primary focus [countries and/or cities]	Early/direct health impacts	Secondary/ societal impacts	State response	Civil society response	Cited in which footnote?
**Asia**									
How Does Covid-19 Affect Urban Slums? Evidence from Settlement Leaders in India	Auerbach, A and T Thachil	n.d.	Institutional grey literature	India, Bhopal, Jaipur				•	30, 31
Ground Report: Chaos at Anand Vihar as Buses Prepare to Take Migrant Workers Home	Ashirwad Mahaprashasta, A and A Srivas	28-Mar-20	News/media	India, Delhi		•			40
Mumbai Kabrastan distributing food supplies at Dharavi	Mumbai Mirror	06-Apr-20	News/media	India, Mumbai				•	41
COVID-19 tech will expand surveillance state in China	Emerald Insight	15-Apr-20	Blog/opinion – institutional	China		•	•		11
Blame It on COVID-19: Domestic Violence on The Rise, Are Men Putting More Pressure on Women?	Deb Roy, L	20-Apr-20	News/media	India		•			80
Covid-19 and the Hrishipara Diarists: was April the cruellest month?	Global Development Institute	11-May-20	Blog/opinion – institutional	Bangladesh		•			70
Responding to COVID-19 in a high-density low-income district in Mumbai	Manivanan Devandra, S (Mahila Milan)	13-May-20	Blog/opinion – institutional	India, Mumbai				•	29
India made its contact tracing app mandatory. Now people are angry	Christopher, N	14-May-20	News/media	India		•	•	•	14

Community-led COVID-19 response: the work of the Philippines Homeless People's Federation	Carampatana, T and R Tuazon (Homeless People's Federation of the Philippines – PACSII)	09-Jun-20	Blog/opinion – institutional	Philippines				●	32
India witnesses upsurge in Covid deaths	South Asia Monitor	22-Jun-20	News/media	India	●				7
How Dhaka's urban poor are dealing with COVID-19	Taylor, J	01-Jul-20	Blog – personal	Bangladesh		●			73
**Africa**									
Corona Diaries of the Urban Poor	JEI	n.d.	Institutional grey literature	Pan-Africa		●		●	65, 66, 69, 70
Covid-19; Reflections from Mukuru's Community Health Volunteers (CHVs)	Adobe Spark	n.d.	News/media	Pan-Africa				●	19
Impact of Covid19 on Lagos Informal Settlements & Vulnerable Urban Poor Populations: Report on Findings	PCEI, NSIFF, JEI	n.d.	Institutional grey literature	Nigeria		●	●	●	68
Know Your City TV, COVID-19	Muungano Alliance	n.d.	News/media	Kenya		●			65
Muungano wa Wanavijiji COVID-19 response	Muungano Alliance	n.d.	Institutional grey literature	Pan-Africa		●		●	65
Regulations and Guidelines - Coronavirus COVID-19	South African government	n.d.	Institutional grey literature	South Africa			●		82
29 areas in SA identified for de-densification to combat coronavirus	BusinessTech	25-Mar-20	News/media	South Africa	●		●		4
Covid-19: Civil society bodies warn against de-densification of informal settlements	Ntseku, M	21-Apr-20	News/media	South Africa, Cape Town		●			52
Zimbabwe faces malaria outbreak as it locks down to counter coronavirus	Chingono, N	21-Apr-20	News/media	Zimbabwe		●			43
Township lockdown: How South Africa's poor bear the cost of coronavirus	Oliver, G	23-Apr-20	Blog/opinion – institutional	South Africa, Cape Town		●			67
Aerial Footage Shows Miles-Long Queue for Food Aid in South Africa	Reuters	30-Apr-20	News/media	South Africa, Pretoria		●			42
At least 5,000 people in Nairobi were left homeless and at risk of contracting COVID-19 after slum demolitions	Bhalla, N	06-May-20	News/media	Nairobi		●			17
'I realised my body was burning': police brutality in Uganda lockdown	Hayden, S	28-May-20	News/media	Uganda,		●			79

Just Empower	JEI	30-May-20	Institutional grey literature	pan-Africa		●			78
27–28 May 2020: coronavirus situation tracker for Kenyan informal settlements	Muungano Alliance	30-May-20	Blog/opinion – institutional	Kenya	●				64
Kenyan police 'killed 15' since start of coronavirus curfew	Al Jazeera	06-Jun-20	News/Media	Kenya		●	●		26
Covid-19 isolation centers: Muungano Alliance contributes to government guidelines	Muungano Alliance	17-Jun-20	Blog/opinion – institutional	Kenya				●	45
19–20 June 2020: coronavirus situation tracker for Kenyan informal settlements	Muungano Alliance	23-Jun-20	Blog/opinion – institutional	Kenya	●				57
Fighting a cholera outbreak in the middle of a COVID-19 pandemic	Taremwa, A	23-Jul-20	Blog/opinion – institutional	Uganda		●		●	43
**Latin America and the Caribbean**									
Latin America's Prison Gangs Draw Strength from the Pandemic	Dudley, S	05-May-20	News/media	Brazil		●			76
Coronavirus in the Daily Life of Favelas, Part 8: In São Paulo, Guaianases Confronts Advancing Pandemic	Veloso, L	06-May-20	News/media	São Paulo		●			69
COVID-19 in Guayaquil: from global notoriety to family responses	IIED	20-May-20	Blog/opinion – institutional	Guayaquil		●			54
Argentina cordons off virus-hit slum as critics decry 'ghettoes for poor people'	Goñi, U	27-May-20	News/media	Argentina		●			77
How are Peru's street vendors facing COVID-19?	Ogando, A C and O Abizaid	11-Jun-20	Blog – personal	Peru		●			35, 38, 46
Coronavirus surges in Latin America as deaths surpass 100,000	Jorgic, D	24-Jun-20	News/media	Pan-Latin America	●				6
Data Favela Study: 80% of Favela Families Are Living on Less than Half of Their Pre-Pandemic Income	RioOnWatch	03-Jul-20	Blog/opinion – institutional	Rio de Janeiro		●			72
Epidemiological Alert: Dengue in the context of COVID-19	PAHO/WHO	28-Jul-20	Institutional grey literature	Pan-Latin America		●	●		43
**Multi-country/multi-region**									
Government responses to COVID-19 crisis	WIEGO	n.d.	Institutional grey literature	Multi-country/ region			●		34
Technical Considerations in Analysing & Advocating for Cash Benefit Schemes for Informal Workers during COVID-19	WIEGO	n.d.	Institutional grey literature	Multi-country/ region		●	●		37
World COVID-19 Stats	N/A	n.d.	Institutional grey literature	Multi-country/ region	●				8

Outbreaks like coronavirus start in and spread from the edges of cities	Keil, R, C Connolly and S Harris Ali	18-Feb-20	Blog – personal	Multi-country/ region		●			3
Lockdowns around the world bring rise in domestic violence	Graham-Harrison, E, A Giuffrida, H Smith and L Ford	28-Mar-20	News/media	Multi-country/region		●			80
UN-Habitat COVID-19 Response Plan	UN-Habitat	Apr-20	Institutional grey literature	Multi-country/ region		●		●	1
From Kenya to Bangladesh mask-making has become a thriving cottage industry	Hodal, K	25-May-20	News/media	Multi-country/ region		●			44
Managing COVID-19 on an increasingly urbanised planet	Connolly, C	26-Jun-20	Blog – personal	Multi-country/ region		●			63
Coronavirus tracked: the latest figures as countries reopen	Financial Times	12-Aug-20	News/media	Multi-country/ region	●				48, 53
**Others**									
COVID-19: Resources to address gender-based violence risks	GBVguidelines.org	n.d.	Institutional grey literature	Other [N/A]		●			80
Pandemics Are Also an Urban Planning Problem	Klaus, I	07-Mar-20	Blog/opinion – institutional	Other		●			63
Density Is New York City's Big 'Enemy' in the Coronavirus Fight	Rosenthal, B	23-Mar-20	News/media	Other [New York]		●			61
The Digital Response to the Outbreak of COVID-19	McDonald, S	30-Mar-20	Blog/opinion – institutional	Other [N/A]		●			10
Germany Says It Has Identified the 1st Coronavirus Transmission in the Country	Schmitz, R B	21-Apr-20	News/media	Other [Germany]	●				2
The COVID-19 crisis: income support to informal workers is necessary and possible	Alfers, L, R Moussié and J Harvey	22-Apr-20	Blog – personal	Other		●		●	37
COVID-19: Exceptional measures should not be cover for human rights abuses and violations – Bachelet	UNHRC	27-Apr-20	Institutional grey literature	Other		●			74
Sitting ducks: UK charity sees surge in calls from stalking victims	Batha, E	11-May-20	Blog/opinion – institutional	Other [UK]		●			80
Scientists are drowning in COVID-19 papers. Can new tools keep them afloat?	Brainard, J	13-May-20	Blog personal	Other [N/A]		●			9
Just Because You Can Afford to Leave the City Doesn't Mean You Should	Bassett, M T	15-May-20	News/media	Other [New York]		●			49
For World's Street Vendors, Life May Never be the Same after COVID-19	Balbuena, P and C Skinner	07-Jun-20	Blog – personal	Other		●			39
Emerging lessons from community-led COVID-19 responses in urban areas	IIED	25-Jun-20	Institutional grey literature	Other		●		●	47
